# Enhancement of lateral flow assay performance by electromagnetic relocation of reporter particles

**DOI:** 10.1371/journal.pone.0186782

**Published:** 2018-01-08

**Authors:** Maria João Jacinto, João R. C. Trabuco, Binh V. Vu, Gavin Garvey, Mohammad Khodadady, Ana M. Azevedo, Maria Raquel Aires-Barros, Long Chang, Katerina Kourentzi, Dmitri Litvinov, Richard C. Willson

**Affiliations:** 1 iBB—Institute for Bioengineering and Biosciences, Department of Bioengineering, Instituto Superior Técnico, Universidade de Lisboa, Lisbon, Portugal; 2 Department of Chemical and Biomolecular Engineering, University of Houston, Houston, Texas, United States of America; 3 Center for Integrated Bio & Nano Systems, University of Houston, Houston, TX, United States of America; 4 Department of Bioengineering, Instituto Superior Técnico, Universidade de Lisboa, Lisbon, Portugal; 5 Department of Electrical & Computer Engineering, University of Houston, Houston, TX, United States of America; 6 Department of Biology and Biochemistry, University of Houston, Houston, Texas, United States of America; 7 Tecnológico de Monterrey, Departamento de Biotecnología e Ingeniería de Alimentos, Centro de Biotecnología FEMSA, Monterrey, Nuevo León, Mexico; The Ohio State University, UNITED STATES

## Abstract

Lateral flow assays (LFAs) are a widely-used point-of care diagnostic format, but suffer from limited analytical sensitivity, especially when read by eye. It has recently been reported that LFA performance can be improved by using magnetic reporter particles and an external magnetic field applied at the test line. The mechanism of sensitivity/performance enhancement was suggested to be concentration/retardation of reporter particles at the test line. Here we demonstrate an additional mechanism of particle relocation where reporter particles from the lower depths of the translucent LFA strip relocate to more-visible locations nearer to the top surface, producing a more visible signal. With a magnetic field we observed an improvement in sensitivity of human chorionic gonadotropin (hCG) detection from 1.25 ng/mL to 0.31 ng/mL. We also observed an increase of the color intensity per particle in test lines when the magnetic field was present.

## Introduction

Lateral flow assays (LFAs) are rapid, simple, and inexpensive point-of-care diagnostic tools which have been used in the detection of a variety of targets such as toxins, pathogens, drugs, and hormones. The home pregnancy test for human chorionic gonadotropin (hCG) is the best-known application of the LFA technology [1]. In a sandwich LFA, a sample wicks by capillary action along a porous chromatographic membrane in which immobilized analyte-specific recognition elements, e.g. antibodies or DNA/RNA probes, form analyte capture test lines. Reporter particles bearing their own recognition elements are analyte-bridged to the membrane at the test line to produce a visible line indicating a positive result. Excess reporter particles are captured in an analyte-independent way at a control line to confirm the proper flow of the liquid along the membrane [1, 2]. A variety of particles such as colloidal gold, colored latex particles, carbon nanoparticles [3], phosphors [4], virus particles [5–7] or magnetic particles [8–18] can be used as reporters.

Magnetic particle reporters may be detected visually by the naked eye [8–10], or by their magnetic properties using magnetic sensors [10–19]. Detection of magnetic particles by their magnetic properties allows for quantitation, although a specialized reader is required. For example, Xu *et al*. described a highly-sensitive lateral flow assay using 111 nm superparamagnetic nanoparticles and a magnetic assay reader, with a detection limit of 0.01 ng/mL cardiac troponin I, compared to 10 ng/mL detectable by enzyme-linked immunosorbent assay (ELISA) [14]. Orlov *et al*. reported that the limit of detection for prostate specific antigen using their magnetic LFA-based detection platform was four times better than for a conventional ELISA, using the same antibody pair [19].

Magnetic particle-based assays also have been proposed in which magnetic particles are used not as reporters, but as capture agents for target/reporter complexes. A magnetic field is applied to concentrate the magnetic particle/target/reporter complexes at the desired location for detection [20, 21]. Although not in LFAs, the application of an external magnetic field also has been shown to modulate magnetic particle movement in a microfluidic channel [22] and to enhance particle binding efficiency and thus assay sensitivity [23–27].

Recently Ren *et al*. (2016) demonstrated that the limit of detection (LoD) of an LFA using magnetic particle reporters could be improved by positioning a permanent magnet under the test line, and suggested that this was because of a longer target-capture line interaction time, leading to increased reporter capture at the detection zone [28]. It also has previously been shown that delaying the sample flow in gold particle LFAs using strips with incorporated hydrophobic barriers [29] or focusing target-gold reporter complexes into a thin band and transporting them to the test line using isotachophoresis [30] can improve the LoD of gold particle LFAs.

Here we present an investigation of the mechanisms of magnetic LFA enhancement sensitivity. By optimizing electromagnet pulse duration, operation synchrony, and location, we test specific hypothesized mechanisms of LFA enhancement by magnetic fields, including increased time of transit of reporter particles through capture zones, and relocation of particles to shallower, more-visible depths in the LFA strip. We find that in the system tested, particle relocation to more visible depths plays an important, previously-unsuspected role in magnetic enhancement of LFA sensitivity.

## Materials and methods

### Chemicals and biologicals

Tween-20, bovine serum albumin (BSA), human chorionic gonadotropin (hCG; 1 μg = 9.28 IU according to the 3^rd^ International Standard), sodium (meta) periodate (NaIO_4_) and hydroxylamine hydrochloride were purchased from Sigma-Aldrich (St Louis, Missouri, USA). Phosphate-buffered saline (PBS) tablets were purchased from Takara Bio Inc. (Shiga, Japan). Sodium cyanoborohydride was obtained from Thermo Fisher (Waltham, Massachusetts, USA). Buffers were prepared using sodium acetate (Mallinckrodt, St Louis, Missouri, USA), sodium carbonate (EM Science, Gibbstown, New Jersey, USA), sodium bicarbonate (EM Science), and pH was adjusted with 1 M sodium hydroxide (Macron, Nashville, Tennessee, USA) and 99.7% min acetic acid (Macron) stock solutions. All buffers were prepared with deionized water (Millipore Milli-Q). Mouse monoclonal anti-β hCG antibody (#ABBCG-0402), polyclonal anti-α hCG antibody (#ABACG-0500) and anti-mouse antibody (#ABGAM-0500) were purchased from Arista Biologicals (Allentown, PA, USA).

### Functionalization of magnetic particles with anti-hCG antibodies

Fc-directed immobilization of the mouse monoclonal anti-β hCG antibodies onto magnetic particles was carried out using periodate-based oxidation of the glycosylated Fc residues [31]. Anti-hCG antibodies (0.9 mL, 0.1 mg/mL in 100 mM sodium acetate buffer, pH 5.4) were reacted with 90 μL of 0.1 M NaIO_4_ for 30 minutes at room temperature with gentle mixing, while protected from light. The oxidized antibodies were immediately purified using 100 kDa Amicon Ultra centrifugal filter units (Millipore, Billerica, MA, USA), and stored in 200 mM sodium carbonate buffer, pH 9.6 (“activation buffer”), at 30 μg/mL for further conjugation steps.

Streptavidin-coated magnetic particles (#0321, Ademtech, Pessac, France; 120 nm diameter: 25 μl, ~2x10^10^ particles) were washed in activation buffer, resuspended in 100 μL of activation buffer and incubated with oxidized antibodies for 2 hours at room temperature with gentle mixing. Sodium cyanoborohydride (NaCNBH_3_) solution (5 M NaCNBH_3_ in 1 M NaOH, 15 μL), was then added to the reaction solution and incubated for 30 minutes at room temperature with gentle mixing. Unreacted aldehydes were quenched with 1 M hydroxylamine in activation buffer (75 μL) for 30 minutes at room temperature with gentle mixing. The antibody-functionalized magnetic particles were separated by magnetic separation, washed thrice in PBS and stored at 4°C (~2x10^8^ particles/μL).

### LFA strip preparation

**Fig 1:**
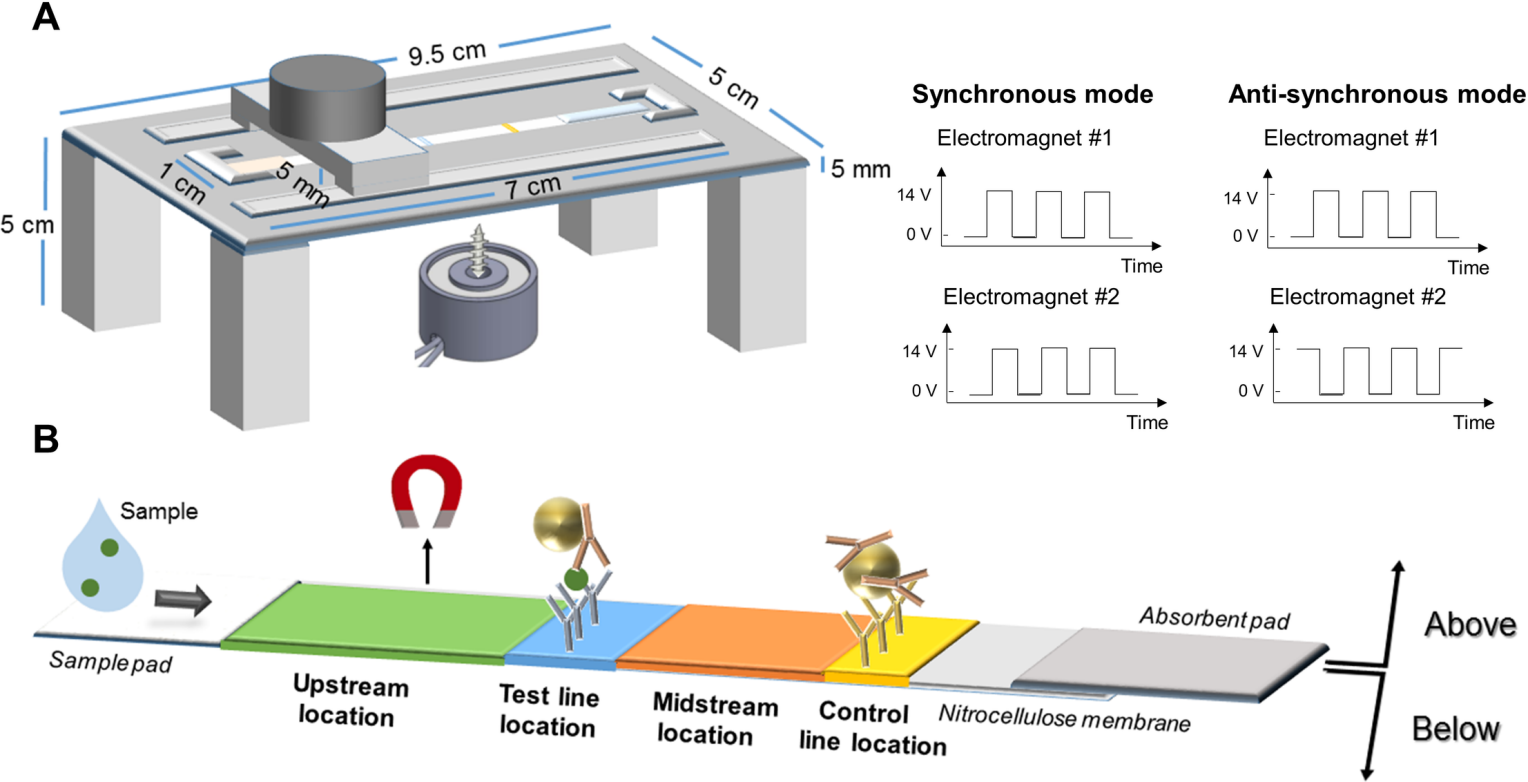
A) 3D-printed assay apparatus. The apparatus holds an electromagnet over the strip’s top surface and also enables the optional placement of another electromagnet below the strip. B) Electromagnet-enhanced LFA for hCG detection. Magnetic particles were functionalized with mouse monoclonal anti-β hCG antibodies. The test line contains goat polyclonal anti-α hCG antibodies, and the control line contains goat anti-mouse antibodies. In this schematic, one electromagnet is shown positioned above the “upstream” location, which is 2.5 cm upstream of the control line and 0.8 cm upstream of the test line. The test line is 1.7 cm upstream of the control line, and the “midstream” location is 1 cm upstream of the control line, between the test and control lines.

Whatman FF80HP nitrocellulose membrane (GE Healthcare) was cut into 6 cm by 29.7 cm pieces. The test and control lines were dispensed using a lateral flow reagent dispenser (Claremont BioSolutions, Upland, CA) powered by a programmable DC power supply (BK Precision 9130) and equipped with an external Fusion 200 syringe pump (Chemyx, Stafford, TX). Antibody solutions (1 mg/mL polyclonal anti-α hCG antibody and 1 mg/mL of anti-mouse antibody solutions in 1x PBS, pH 7.4) were dispensed at a rate of 0.22 mL/min with a head speed of 4 cm/s, providing a dispensed protein quantity of 1 μg/cm. The lines were spotted 1.7 cm apart from each other, and the test line was spotted at 1.3 cm from the sample pad end (Fig 1). The membranes were assembled on adhesive cards from DCN Diagnostics with Fusion 5 sample pads and Whatman CF5 absorbent pads. The membrane assembly was then cut using a standard paper guillotine, to create individual strips 4 mm wide and 7 cm long. The strips were then allowed to dry overnight at room temperature and stored desiccated until use.

### Lateral flow assay–optimization tests

hCG test protein was diluted in PBS and 50 μL of each sample was dispensed onto the sample pad of strips with anti-hCG antibodies on the test line and anti-mouse antibodies on the control line. The strips were washed with 20 μL LFA buffer (1% Tween-20, 0.5% BSA, in PBS, pH 7.4) before applying 5 μL of the conjugated magnetic beads (~10^9^ particles). The strips were washed again with 200 μL LFA buffer and immediately imaged using a flatbed color scanner (Perfection V600, Epson, Long Beach, CA). The scanned images were analyzed with the Gel Analysis Tool of ImageJ (National Institutes of Health, Bethesda, MD, USA) [32] by plotting the line intensity profile and numerically integrating the area under each peak. Ratios of the test line (T) intensity to the control line (C) intensity, T/C, were calculated for each strip and replicates (n = 3) were averaged.

### Determination of LoD

To evaluate the analytical sensitivity of the assay, “half-strip” dipstick LFAs (3 mm wide, consisting only of membrane and absorbent pad, with use of sample and conjugate pads avoided for maximal reproducibility) were performed. 50 μl of hCG test protein in PBS ranging from 1.25 ng/mL down to 0.03 ng/mL (serial two-fold dilutions), were incubated with 1 μL of the conjugated magnetic beads (~10^9^ particles) for 5 min. The sample was then concentrated to 10 μL using a magnet. The half-strip was dipped into the sample for 10 min. The strips were then washed with 50 μL LFA buffer (1% Tween-20, 0.5% BSA, in PBS, pH 7.4). The strips were dried for 2 hours, then scanned, and images were processed as described in ESI. The limit of detection was determined as the lowest concentration measured to be above the mean plus 3 times the standard deviation of the no-analyte control.

### Electromagnet apparatus and programming

For magnetically enhanced LFA, two cylindrical electromagnets, 1.75” diameter X 1.25” height, (EM175-12-222, APW Company, Rockaway, NJ) were fitted with a pointed 2.5 cm length, 5 mm steel core to increase the field gradient and to concentrate the magnetic force on a specific area. A custom LabView user interface was used to modulate the operating duration and timing of the electromagnets. The system can operate the two electromagnets independently (in unsynchronized mode) or in concert (synchronized mode). In synchronized mode, the electromagnets can be switched on or off simultaneously (synchronous mode) or alternatively (anti-synchronous mode).

To ensure reproducibility, a 3D-printed structure was used to fix the location of the electromagnets along the strip. The electromagnets were placed 5 mm above or below the strip, providing 0.03 Tesla magnetic field (measured using a Cole-Palmer 5170 Gaussmeter) to the strip at that location. Electromagnets could be placed between the sample pad and test line, at the test line, between the test and control lines, or at the control line (Fig 1). A modified 3D-printed structure compatible with the half-strip dipsticks was also designed for LoD tests (S1 Fig). The apparatus holds the electromagnets over the half-strip’s top and bottom surfaces and the positions can be adjusted and locked by a nut and bolt system. Two glass slides are glued perpendicular to the magnets to sandwich the membrane between them. An opening in the bottom part of the apparatus fits a liquid container, made by cutting a 2 mL microcentrifuge tube at the 0.5-mL line. The apparatus is used in a “vertical” orientation with the TOP side of the membrane on the left and the BOTTOM side on the right. Fluids in the liquid container are exchanged either by replacing the container with a new one or by manual pipetting.

### Magnetic particle counting by Alternating Gradient Field Magnetometer (AGFM)

A magnetometer was used to estimate the total number of magnetic material accumulated on the control and test lines. The control and test lines were cut out using a guillotine. An extra margin of 1 mm upstream and downstream of the position of each line was cut to ensure that the entire line was included in the resulting 3 mm x 3 mm cut out square. Each individual square was placed in a previously calibrated quartz AGFM probe, and a set of gradient coils were used to generate an alternating magnetic field that caused the probe to vibrate. The amplitude of such vibration (measured as electric signal generated by a piezo electric device) is proportional to the magnetic moment of the sample. The number of particles (n_particles_) was determined from the measured magnetic signal (magnetization) with AGFM:

$$n_{particles}=\frac{M_{T}}{M_{s}\times \rho \times V}$$

Where M_T_ is the total magnetization measured by AGFM, M_s_ is magnetization at saturation of particles (40 emu/g), ρ is particle density (2 g/cm^3^), and V is particle volume (4.2x10^-12^ mm^3^).

## Results and discussion

In this work, we investigated two mechanisms of enhancement of magnetic lateral flow assays: (1) the mechanism of particle retardation, where a magnetic field is used to slow down the passage of the reporters through the test line and increase the specific binding, and (2) the mechanism of particle relocation, where a magnetic field is used to alter the location of magnetic particles in the lateral flow membrane towards the upper, more visible, depths of the membrane. Using two electromagnets, we determined the optimal operating mode (timing and magnet position) with a model LFA using anti-mouse antibody test lines and magnetic particles modified with mouse antibodies. Furthermore, we demonstrated an increase in sensitivity of an hCG lateral flow assay.

### Particle retardation

To confirm the effect of magnetic forces on retarding the movement of particles along the membrane, the particle transit time in a 6-cm unmodified Whatman FF80HP membrane (from sample application to visually-observed arrival of particles at the absorbent pad) was measured in the presence and absence of the magnetic field. Without the magnetic field, the transit took 22 ± 2 minutes (n = 3); When the electromagnets were applied (10-second on/10-second off pulses at 14 V, 0.03 Tesla field strength, electromagnets at top upstream and bottom midstream positions in unsynchronized mode) the transit time increased from 22 to 28 ± 2 minutes (n = 3), confirming that electromagnets can retard the movement of the particles, as previously suggested [28].

### Pulse duration effect on test performance

**Fig 2:**
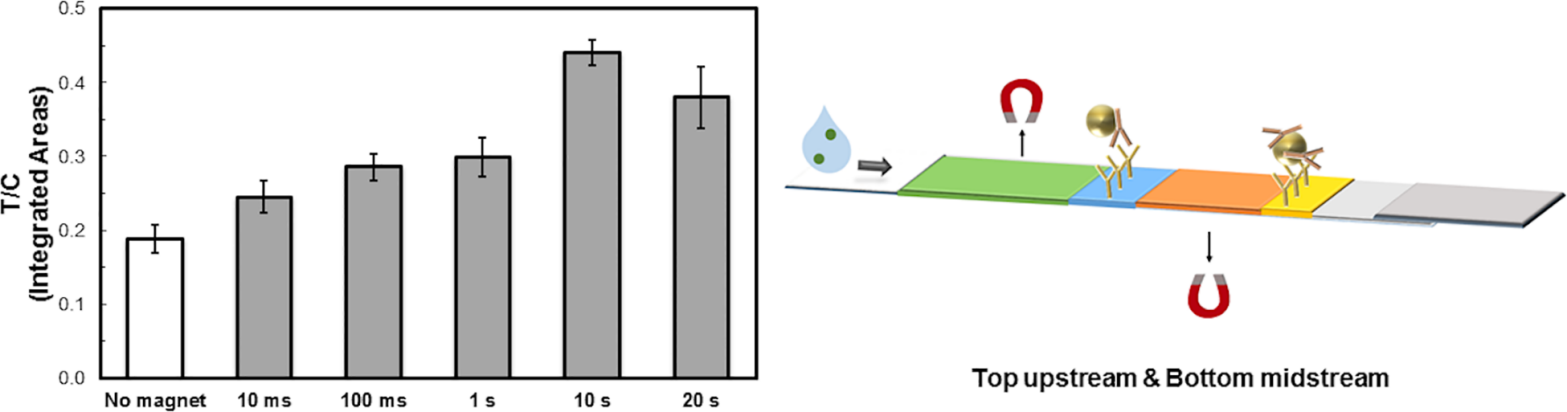
Effect of pulse duration at constant 50% duty cycle on electromagnetically controlled LFA performance factor. 13.5 ng/mL hCG was detected using anti-hCG antibodies at the test line and mouse monoclonal anti-β hCG antibodies-functionalized magnetic particles. Control line consisted of anti-mouse antibodies. Two electromagnets were applied in top upstream and bottom midstream positions with different pulse durations at 14 V, in unsynchronized mode. For example, 10 ms represents 10-millisecond on/ 10-millisecond off pulses, unsynchronized. Line intensity profiles were evaluated by ImageJ density analysis [32]. The area under each peak was numerically integrated using the ImageJ Gel Analysis Toolbox, and the ratio of the test line intensity (T) divided by the intensity of the control line (C) for each strip was calculated, then averaged (n = 3, mean ± SD).

On/off pulses of varied durations were applied while holding the fraction of time that the magnets were active (“duty cycle”) constant at 50%. In these experiments, the electromagnetic apparatus was operated in the unsynchronized mode with two electromagnets above the upstream (0.8 cm before the test line; 2.5 cm before the control line) and below the midstream position (1 cm upstream of the control line) as illustrated in Fig 2. We observed that the assay performance was significantly affected by varying the magnetic pulse duration while keeping duty cycle unchanged (50%).

As the magnetic pulse duration increased from 10 ms to 10 s, the T/C ratio of test line signal to control line signal steadily increased, from 30% improvement at 10 ms to 234% improvement at 10 s, when compared to control test with no magnet. We observed that a 20-s pulse duration was less effective compared to a 10-s pulse duration. We speculate that there is a tradeoff between relocation to the surface with greater visibility, and spreading the particles among the available antibody binding sites (as opposed to over-saturating the antibody sites at the very top). The total transit time for the particles to travel the length of the LFA strip was not significantly affected by pulse duration, at constant 50% duty cycle. This suggests that simple magnetic force retardation (slower passage of magnetic reporter particles through test lines increases capture efficiency) is not the sole mechanism of magnetic LFA enhancement as indicated by prior published work [28].

### Particle relocation to more-visible depths

**Fig 3:**
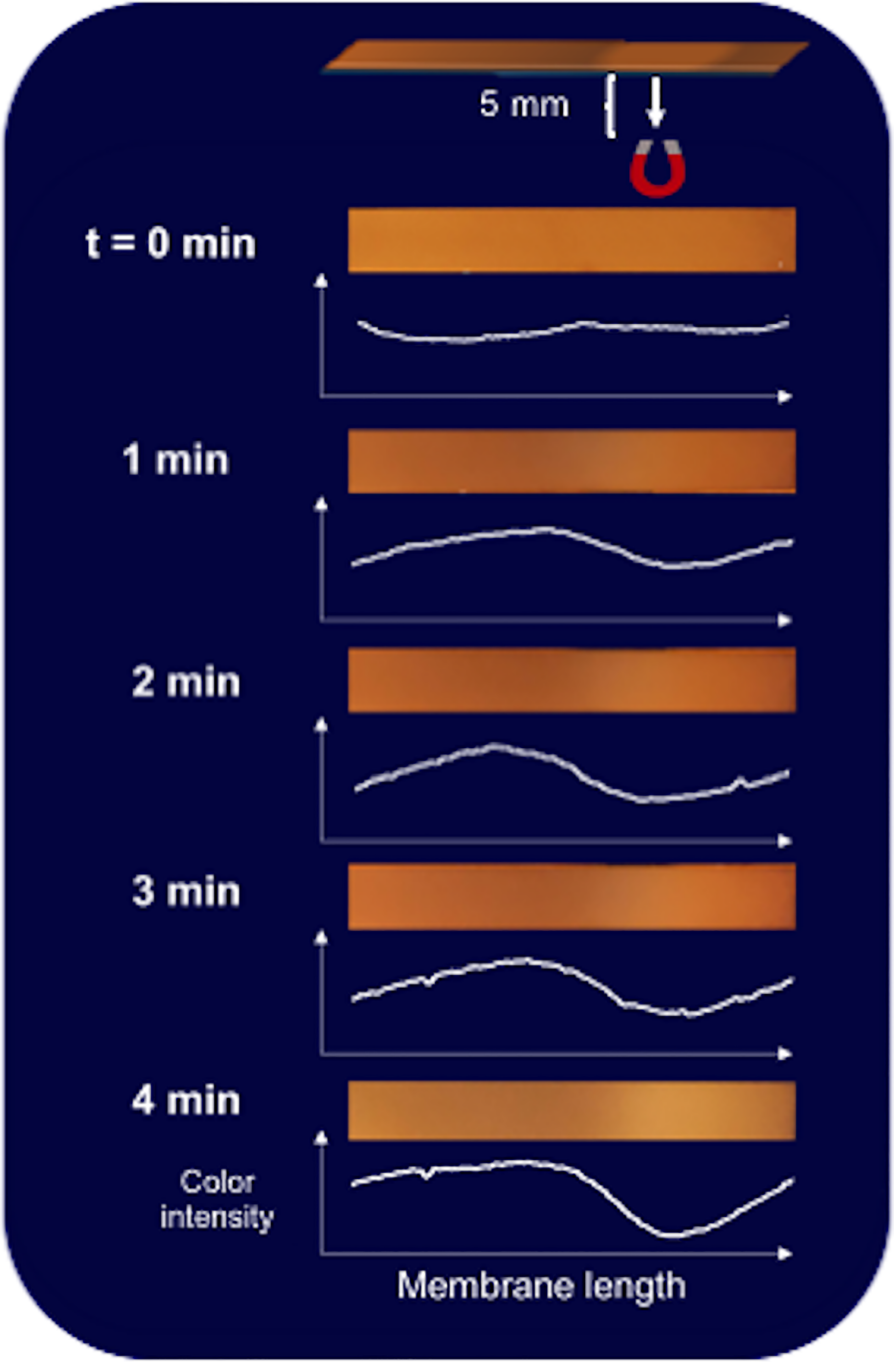
Vertical magnetic particle movement under the influence of an electromagnet. Images at 1 min intervals from above a particle-impregnated LFA membrane with an electromagnet applied 5 mm below the strip at the midstream position (10-second on/10-second off pulses at 14 V, 0.03 Tesla). Image intensity profiles using the same ImageJ image analysis settings and axes for each image. The electromagnet position is indicated in the top schematic.

We hypothesized that magnetic forces can relocate particles closer to the surface of the translucent membrane, increasing their visibility and the resulting LFA signal strength. The apparatus design and opaque backing of the nitrocellulose membranes used precluded imaging the particles when applying an electromagnet above the strip. An electromagnet was therefore positioned 5 mm below a particle-impregnated nitrocellulose membrane (Fig 3), and the gradual “whitening” of the surface of the strip was observed as a measure of particle mobility and relocation.

As shown in Fig 3, when an electromagnet was applied below the strip the particles gradually moved deeper into the translucent strip where they became less visible, leaving a lighter color on the top surface. This suggests that an electromagnet above a typical 100-μm thick LFA membrane can influence the vertical flow of particles throughout the thickness of the strip, by dragging the particles towards the more-visible top of the membrane, and thus enhancing optical detection. In a related experiment, magnetic particles were applied to the sample pad and allowed to wick across the width of the strip with an electromagnet placed at the side of the LFA strip (S2 Fig). We observed the movement of the majority of the particles across the width of the membrane, and the darkening of the side of the strip nearest to the electromagnet. This confirmed that the electromagnet can magnetophoretically move particles even when located 5 mm from the edge of the strip.

### Electromagnetic modulation of capture line intensity

**Fig 4:**
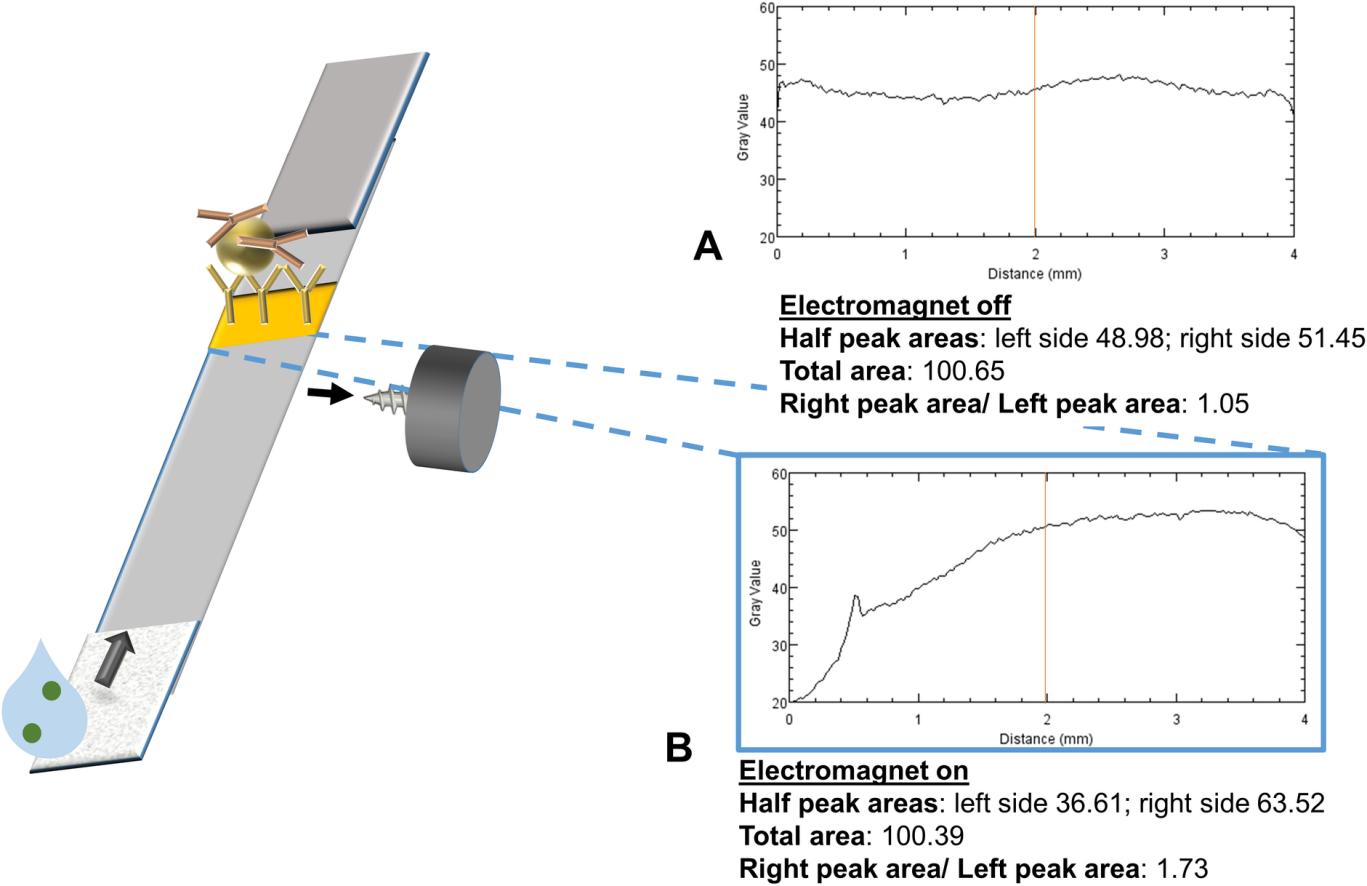
Control line intensity profile in the absence (A) or presence (B, 10 s pulse duration at 50% duty cycle, 0.03 Tesla) of an electromagnet at the right midstream position.

In order to investigate the effect of the electromagnet on signal intensity, ~10^9^ magnetic particles, modified with mouse monoclonal anti-β hCG antibodies, were applied to strips with a single capture line of goat anti-mouse antibodies and an electromagnet was applied on the right side of the strip at the midstream position (10 s pulse duration at 50% duty cycle, 0.03 Tesla) (Fig 4). The resulting capture line intensity profile had the same integrated total intensity but was strongly shifted toward the magnet, as compared to that of an identical strip run without an electromagnet. This result further supports the idea that magnetic forces can induce significant repositioning of magnetic particles within LFA strips.

### Redistribution after magnetic relocation

**Fig 5:**
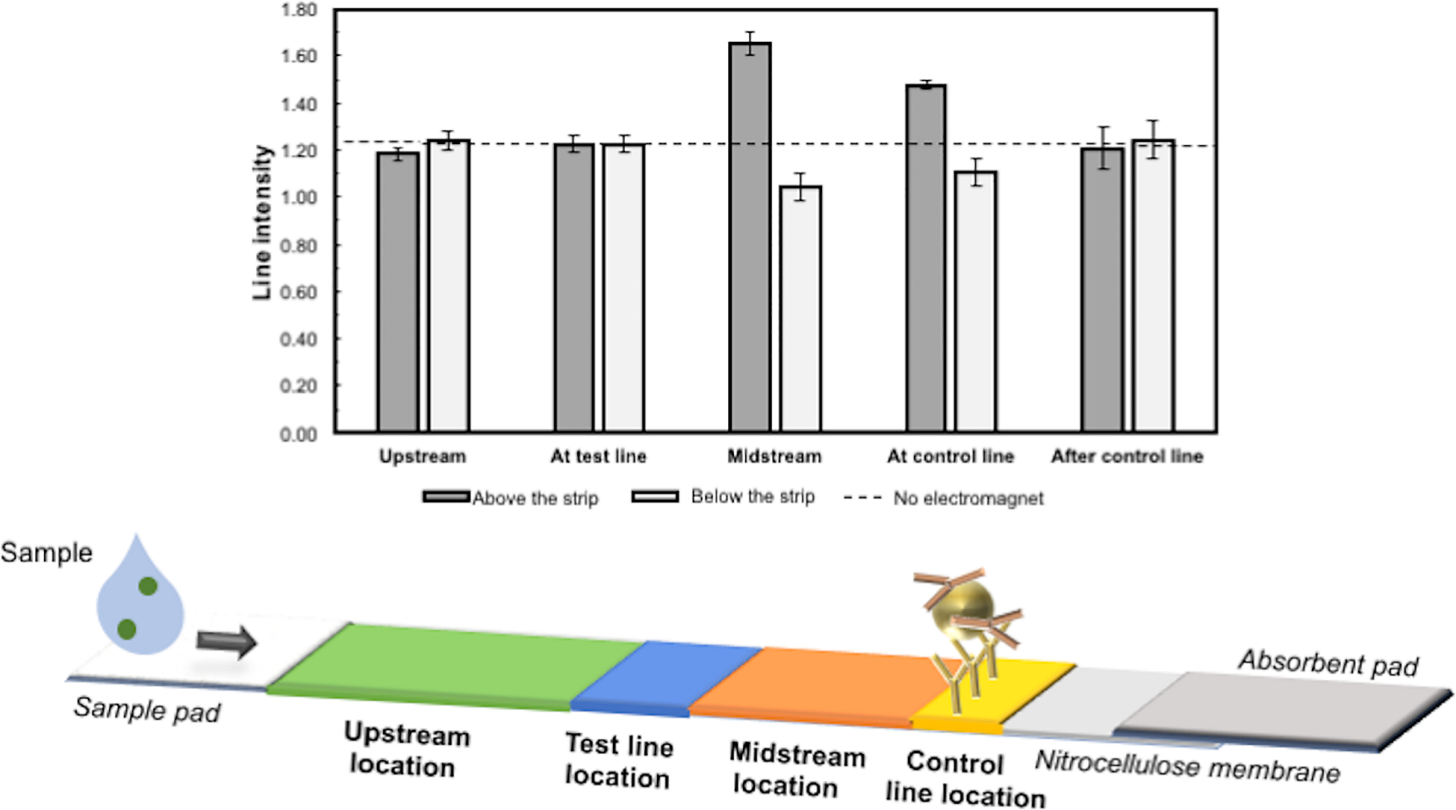
Average control line intensity in magnetic LFA for different positions of a single electromagnet. Magnetic particles modified with mouse monoclonal anti-β hCG antibodies were tested for binding to an anti-mouse antibody control line located in the same position as a typical control line. A single electromagnet was applied at different positions with 10 s pulse duration at 50% duty cycle and 0.03 Tesla field strength. “Above/below the strip” corresponds to the electromagnet positioned 5 mm above or below the LFA strip. Line intensity profiles were evaluated by ImageJ density analysis. The area under each peak was numerically integrated using the ImageJ Gel Analysis Toolbox and replicates averaged. The horizontal dashed line shows the intensity of the line in the absence of the electromagnet (n = 3, mean ± SD; line intensity for no magnet control: 1.23 ± 0.02).

To examine the time-dependence of magnetic repositioning of reporter particles during an LFA, the effect of electromagnet location on capture line intensity was studied using particles modified with mouse monoclonal anti-β hCG antibodies binding to an anti-mouse antibody capture line. All experiments used 10 s pulse duration at 50% duty cycle with 0.03 Tesla field strength. All possible positions of a single electromagnet along the strip (upstream, test line, midstream, control line, and 1 cm after the control line) both above and below the LFA strip were tested (Fig 5).

While prior work addressed the hypothesis that slower passage of magnetic reporter particles through test lines could increase capture efficiency, our results suggest that particle relocation from the deeper and less-visible regions of the membrane also plays an important role in magnetically-enhanced LFA signals. As seen in Fig 5, we observed that applying magnetic forces away from the test and control lines can affect LFA signal intensity even more strongly than magnetic forces applied directly on top of the lines, suggesting an additional mechanism independent of particle retardation in capture zones.

We observed that placing a single electromagnet upstream of or directly on top of the test line location did not affect the intensity of the control line (I_CL_ = 1.18±0.03. vs. I_CL_ = 1.22±0.04, respectively) compared to the no-electromagnet condition (I_CL_ = 1.23 ± 0.02). We believe that this is because of the relatively large distance between these positions and the control line (2.5 and 1.7 cm)—even if the magnetic particles were initially drawn to the top, they could redistribute throughout the membrane thickness before reaching the control line. On the other hand, placing a single electromagnet above the midstream location, 1 cm before the control line, increased its intensity (I_CL_ = 1.65 ± 0.05) compared to the no-electromagnet test (I_CL_ = 1.23 ± 0.02), suggesting that the magnetic particles were pulled by the electromagnet to flow (and bind) closer to the upper surface of the strip, where they are more visible. When applying one electromagnet directly above the control line position, the control line intensity also increased (I_CL_ = 1.48 ± 0.02) but not as much as with a magnet 1 cm upstream. This suggests that the particles need time to magnetophorese towards the surface and become visible. This hypothesis is also supported by the data in Fig 3, which suggest a time scale of several minutes for vertical transport of the particles in the LFA membrane. Magnets placed below the strip were also tested; generally, the results were the inverse of those observed with a magnet above the strip. Finally, an electromagnet placed above or below the strip at 1 cm downstream of the control line produced no significant effect, as expected (I_CL_ = 1.21 ± 0.09 and I_CL_ = 1.24 ± 0.08, respectively). These results together provide further evidence against a retardation-only mechanism of magnetic LFA enhancement.

### Demonstration of enhanced analytical performance of the magnetic LFA

**Fig 6:**
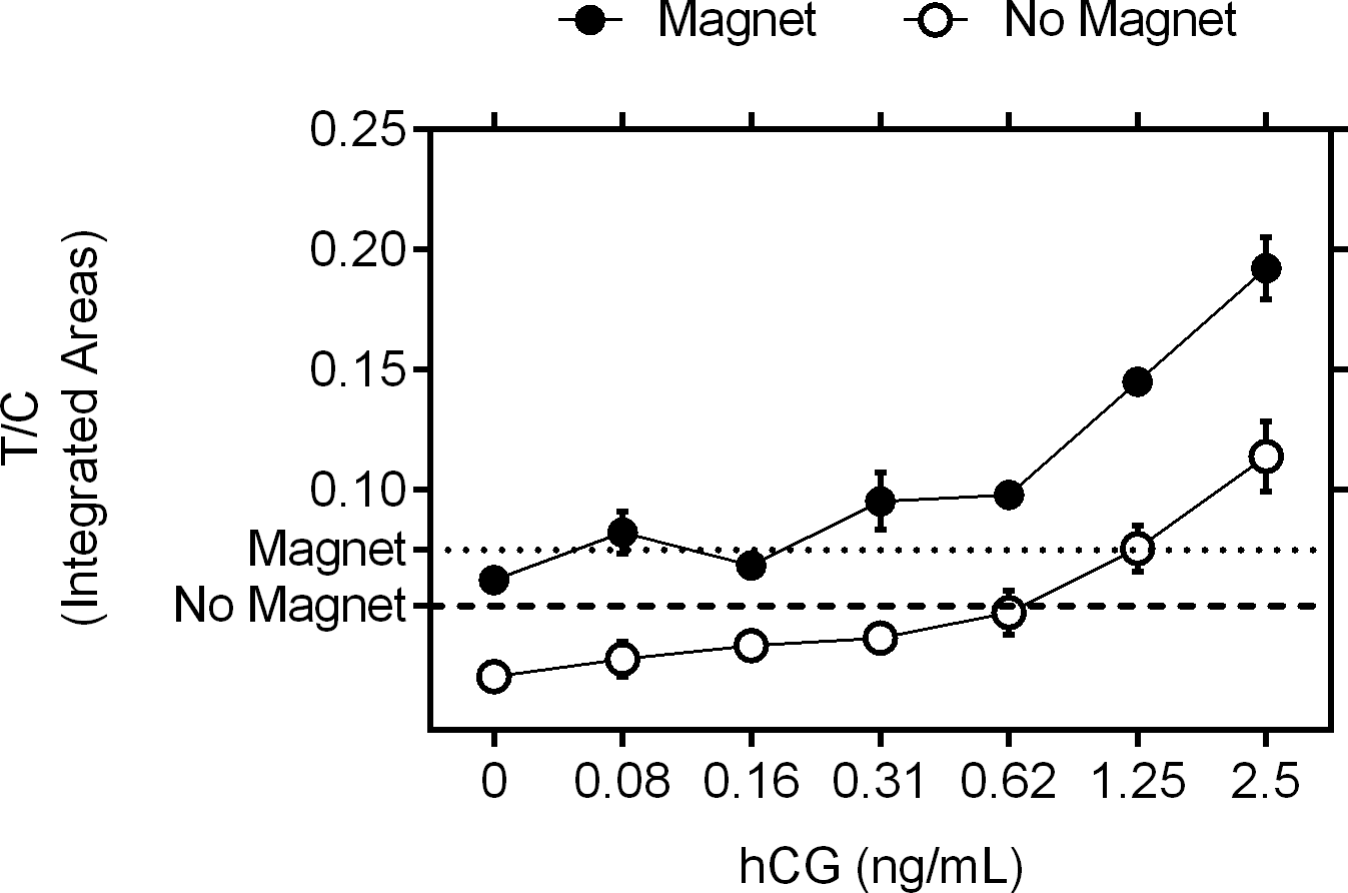
Limit of detection (LoD) of hCG LFA in the presence or absence of the magnetic field. Two-fold hCG dilutions in PBS ranging from 2.5 ng/mL to 0.08 ng/mL were tested in half-strip dipsticks with anti-hCG antibodies as a test line and anti-mouse as a control line in the presence (full dots) and absence of a magnetic field (open dots). The strips were imaged and the T/C ratios of integrated areas of respective peaks were calculated (n = 3). Limit of detection was considered to be the lowest tested concentration above the zero value plus three times its standard deviation (background + 3SD; dotted line denotes signal for the series with magnetic field present and dashed line denotes signal for the series without magnetic field).

We used “half-strip” dipsticks (to maximize throughput and reproducibility), to determine the analytical sensitivity of the magnetically-enhanced LFA when using the optimized magnet setup. The magnetic particles, widely used in sample cleanup and concentration, were also used here to pre-concentrate (by 5-fold) the target assemblies and thus maximize the benefits of magnetic particles before being added to the dipstick. Two-fold serial dilutions of hCG protein ranging from 0.08 to 2.5 ng/mL, were tested using half-strip LFAs in the presence and absence of the magnetic field (10 s pulse duration at 50% duty cycle, 0.03 Tesla, anti-synchronous mode, in three replicates (S5 Fig)). As expected, the T/C ratio increased as the concentration of the hCG protein increased for both groups. Moreover, the LoD, defined as the lowest concentration measured to be above the mean plus 3 times the standard deviation of the no-analyte control, was 0.31 ng/mL in the presence of the magnetic field, compared to 1.25 ng/mL when no magnetic field applied (Fig 6).

### Magnetometric quantitation of bound particles

**Fig 7:**
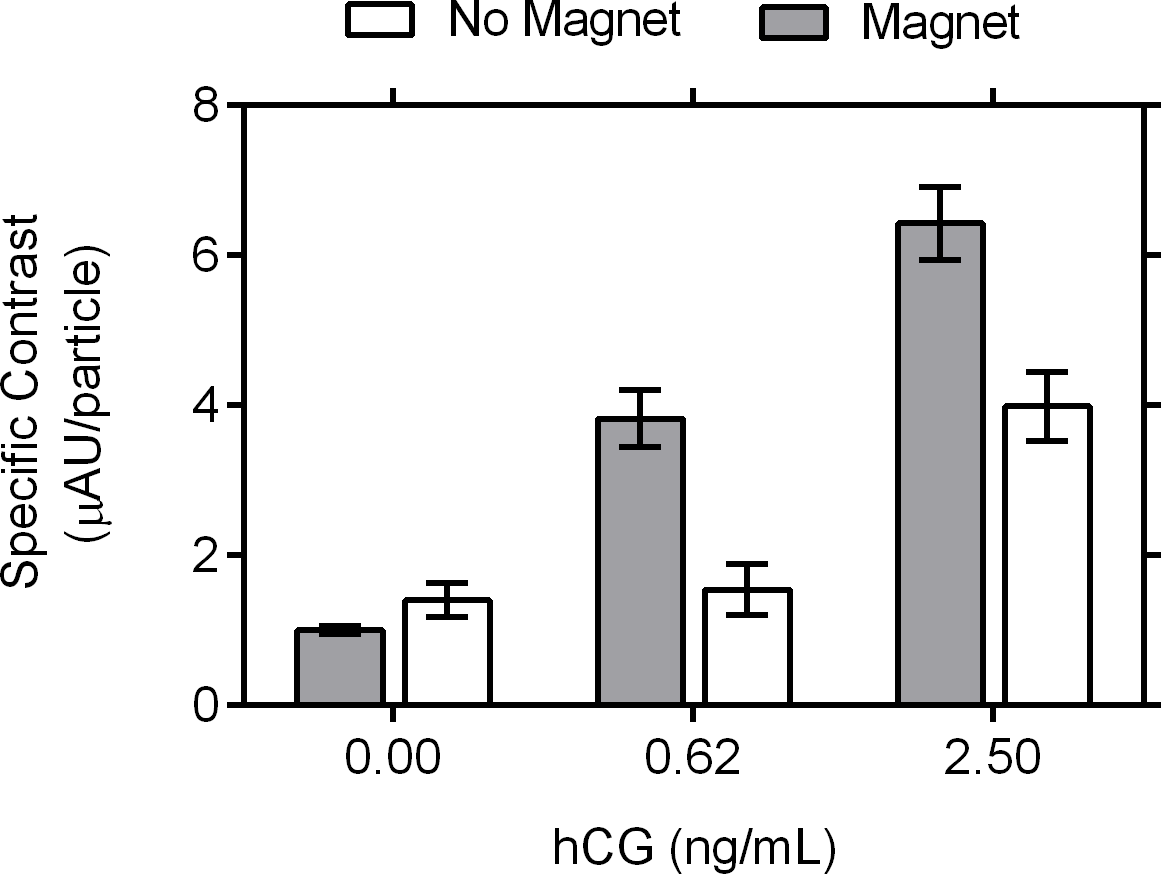
Specific contrast of LFA test line (peak area divided by the total number of particles) in the presence or absence of magnetic field. Specific contrast, the ratio of integrated area of the imaged test line peak to the number of particles measured by AGFM, was calculated for 0, 0.62 and 2.50 ng/mL hCG in assays run with and without a magnetic field, mean and standard error are represented (n = 3). Two-way ANOVA was performed and statistically significant (95%) variance caused by concentration (p<0.0001), magnet (p = 0.0003), and interaction between concentration and magnet (p = 0.028) was found.

The magnetic measurement with AGFM allowed the estimation of the number of magnetic particles captured on the test and control lines. As expected, as analyte concentration increased, the number of particles captured was increased. Additionally, more particles were captured when the magnetic field was present (S7 Fig). The specific contrast, defined as the ratio of the visual signal to the magnetic signal (number of particles) measured by AGFM, was also calculated. As shown in Fig 7, the specific contrast for the test line increases with analyte concentration and the presence of a magnetic field.

The presence of the electromagnet increased the visual signal per particle. Reporters exposed to higher amounts of hCG yield a higher amount of hCG-reporter conjugates that get captured at the test line, enhancing the capacity to produce a visible line (S7 Fig). Moreover, the presence of the magnetic field, allows the formation of a better contrasting test line. Particle relocation initiated in the region immediately before the test line, allows a greater number of conjugates to be captured by the test line. Relocation generates a local concentration effect that is responsible for better particle binding in the visible regions (at the top) of the strip and when combined with the observed particle retardation this results in the observed enhancement of the limit of detection and the visual contrast of test line on the hCG lateral flow assay.

## Conclusions

The application of magnetic field pulses in an LFA test conducted with magnetic particles has been suggested to delay the passage of reporter particles through capture zones, increasing their specific capture. We tested this effect, and confirmed an improvement in LoD from 1.25 ng/mL to 0.31 ng/mL for hCG detection. Electromagnets retard the movement of particles through the capture lines leading to increased capture in that region. We also adduced evidence supporting an alternative mechanism, where magnetic forces bring particles to or near the more-visible top surface of the LFA strip, increasing their specific contrast. We showed that the upstream positioning (1 cm before a capture line) of a magnet is more effective than positioning the magnet at the line and may increase the transport of the particles vertically into the more-visible parts of the LFA membrane.

## Supporting information

S1 Fig 3D-printed structure for the LOD determination tests. (DOCX)

S2 Fig Lateral diversion of flowing particles by electromagnet. (DOCX)

S3 Fig Average control line intensity for different arrangements of two electromagnets. (DOCX)

S4 Fig Effect of electromagnet operation mode on electromagnetically controlled LFA performance factor. (DOCX)

S5 Fig Effect of electromagnetically controlled LFA performance in hCG dilution series. (DOCX)

S6 Fig Image processing for T/C determination and histogram correction. (DOCX)

S7 Fig Number of particles and visual peak area at the test line for hCG dilution series in the presence and absence of the magnetic field. (DOCX)
