# Correction: Enhancement of lateral flow assay performance by electromagnetic relocation of reporter particles

**DOI:** 10.1371/journal.pone.0213859

**Published:** 2019-03-12

**Authors:** Maria João Jacinto, João R. C. Trabuco, Binh V. Vu, Gavin Garvey, Mohammad Khodadadi, Ana M. Azevedo, Maria Raquel Aires-Barros, Long Chang, Katerina Kourentzi, Dmitri Litvinov, Richard C. Willson

The fifth author’s name is spelled incorrectly. The correct name is: Mohammad Khodadadi. The correct citation is: Jacinto MJ, Trabuco JRC, Vu BV, Garvey G, Khodadadi M, Azevedo AM, et al. (2018) Enhancement of lateral flow assay performance by electromagnetic relocation of reporter particles. PLoS ONE 13(1): e0186782. https://doi.org/10.1371/journal.pone.0186782
