# Supplementary material for: Enhancement of lateral flow assay performance by electromagnetic relocation of reporter particles
Source: PLoS One. 2018 Jan 8;13(1):e0186782. doi: 10.1371/journal.pone.0186782 (PMC5757911; doi:10.1371/journal.pone.0186782)
Supplement: S1 Fig — (DOCX) [file pone.0186782.s001.docx]

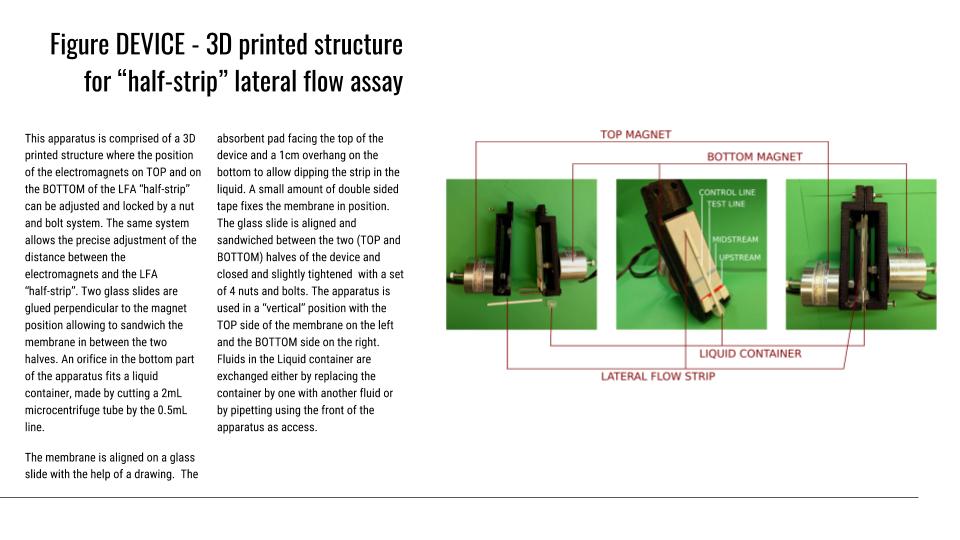


**S1 Fig. 3D-printed structure for the LOD determination tests.** The apparatus holds the electromagnets over the half-strip’s top and bottom surfaces and the positions can be adjusted and locked by a nut and bolt system. Two glass slides are glued perpendicular to the magnets allowing to sandwich the membrane in between them. An opening in the bottom part of the apparatus fits a liquid container, made by cutting a 2mL microcentrifuge tube at the 0.5-mL line. The apparatus is used in a “vertical” orientation with the TOP side of the membrane on the left and the BOTTOM side on the right. Fluids in the liquid container are exchanged either by replacing the container by a new one or by pipetting on the front of the apparatus.
