# Supplementary material for: Enhancement of lateral flow assay performance by electromagnetic relocation of reporter particles
Source: PLoS One. 2018 Jan 8;13(1):e0186782. doi: 10.1371/journal.pone.0186782 (PMC5757911; doi:10.1371/journal.pone.0186782)
Supplement: S2 Fig — (DOCX) [file pone.0186782.s002.docx]

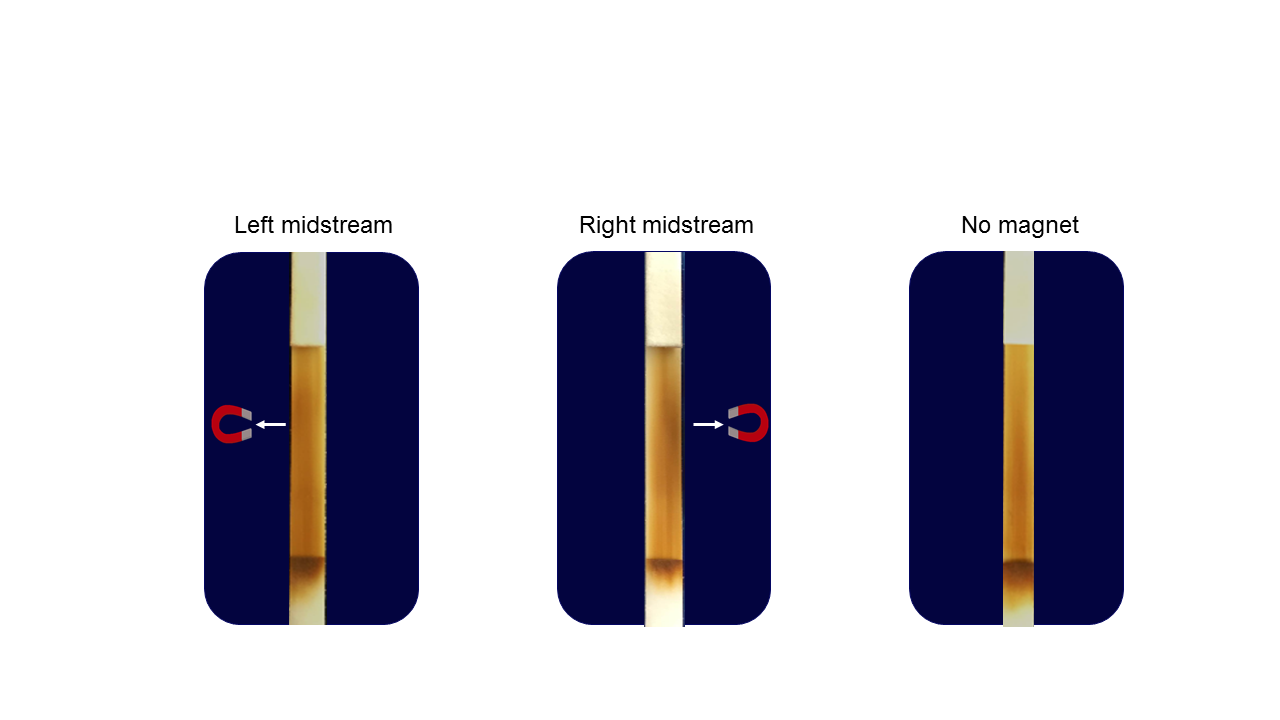


**S2 Fig. Lateral diversion of flowing particles by electromagnet.** Magnetic particles move across the LFA membrane when an electromagnet is positioned on the left or the right midstream location, confirming that the magnet affects the particles even at a significant distance. In the absence of the electromagnet, the majority of the particles flow within the central region of the strip; the particles were applied onto the center of the sample pad. White arrows indicate the magnetic force direction (10-second on/ 10-second off pulses at 14 V, 0.03 T).
