# Supplementary material for: Enhancement of lateral flow assay performance by electromagnetic relocation of reporter particles
Source: PLoS One. 2018 Jan 8;13(1):e0186782. doi: 10.1371/journal.pone.0186782 (PMC5757911; doi:10.1371/journal.pone.0186782)
Supplement: S3 Fig — (DOCX) [file pone.0186782.s003.docx]

**Combined action of two electromagnets**

After investigating the effect of a single electromagnet, the combined application of two electromagnets was studied. Magnetic particles modified with mouse monoclonal anti-β hCG antibodies were tested for binding to anti-mouse antibody control lines, one located in the position of a typical LFA test line (CL#1) and another in the position of a typical LFA control line (CL#2) (S3 Fig). All tests were performed using 10-second on/10-second off unsynchronized pulses at 14 V for the entire assay duration. When no electromagnets were applied, the intensities of the two lines were similar: ICL#1 = 1.34±0.03 and the second-encountered line ICL#2 = 1.27±0.06. Applying one electromagnet at the upstream position above the strip (0.8 cm before the first line and 2.5 cm before the second line) and the other electromagnet at the midstream position (between the two capture lines) below the strip led to an increase of the intensity of the first anti-mouse line (ICL#1 = 1.63±0.05) and a decrease in the intensity of the second (ICL#2 = 0.88±0.07). As we previously hypothesized, an electromagnet applied to a surface of a strip may pull the particles towards the upper surface where they bind and are more visible or down to where they cannot be seen. On the other hand, when the two electromagnets are applied in an opposing arrangement, below upstream (0.8 cm before the first line and 2.5 cm before the second line), and above midstream (1 cm upstream of the first line), the first anti-mouse line intensity decreases (ICL#1 = 0.96±0.03 vs. 1.34±0.03 when no electromagnet is applied) and the second one shows a higher intensity than the first line but lower than its previous no-magnet value (ICL#2 = 1.16±0.02 vs. no-magnet 1.27±0.06). Although the dwell time at the first line increases and more particles could bind at CL#1, they bind towards the bottom part of the strip where they are less visible. This could lead to fewer particles being available and still in a lower strip depth to flow to CL#2 and thus we could not observe the expected (because of the presence of the second electromagnet) increase in the second anti-mouse line.


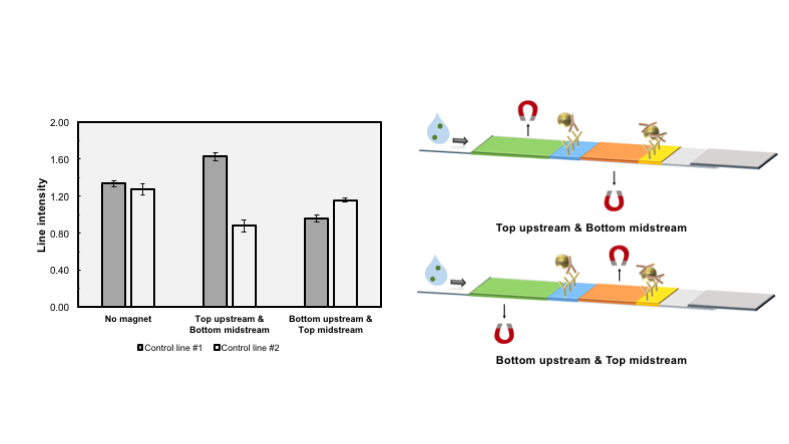


**S3 Fig. Average control line intensity for different arrangements of two electromagnets.** Magnetic particles modified with mouse monoclonal anti-β hCG antibodies were tested for binding to two anti-mouse antibody control lines; control line #1 (CL#1) is located in the position of a typical LFA test line and control line #2 (CL#2) is located in the position of a typical LFA control line. Electromagnets were applied in different arrangements with 10-second on/ 10-second off unsynchronized pulses at 14 V, for the entire assay duration. Line intensity profiles were evaluated by ImageJ density analysis. The area under each peak was numerically integrated using the ImageJ Gel Analysis Toolbox and replicate strips were averaged; (n=3, mean ± SD).
