# Supplementary material for: Enhancement of lateral flow assay performance by electromagnetic relocation of reporter particles
Source: PLoS One. 2018 Jan 8;13(1):e0186782. doi: 10.1371/journal.pone.0186782 (PMC5757911; doi:10.1371/journal.pone.0186782)
Supplement: S4 Fig — (DOCX) [file pone.0186782.s004.docx]

**Electromagnet operation mode**

The combined application of two electromagnets allows three possible modes of operation. As previously described, the electromagnets can be switched on or off at the same time (synchronous mode) or in an alternating fashion, when one electromagnet is on, the other is off (anti-synchronous mode), or randomly in an unsynchronized manner. Two electromagnets were applied in top upstream and bottom midstream positions with 10-second on/ 10-second off pulses at 14 V, in different operation modes (synchronous, anti-synchronous or unsynchronized). The unsynchronized mode showed a 134% increase in the T/C ratio compared to a test with no electromagnets applied, and the anti-synchronous mode resulted in a 90% increase in the T/C ratio (S4 Fig). For both cases, as compared to the no-magnet control, the intensity of the TL increased whereas the intensity of the CL decreased but to a different extent. Despite the observations for a single electromagnet case in Fig 5 (main text), the synchronous mode resulted in no significant increase in the raw intensities of the test and control lines and the T/C performance factor. This was probably due to the fact that the combined and synchronized action from the top upstream and bottom midstream electromagnets created an overlap in the magnetic fields, regions in which the particles are stationary and are moved neither to the top nor to the bottom of the membrane.


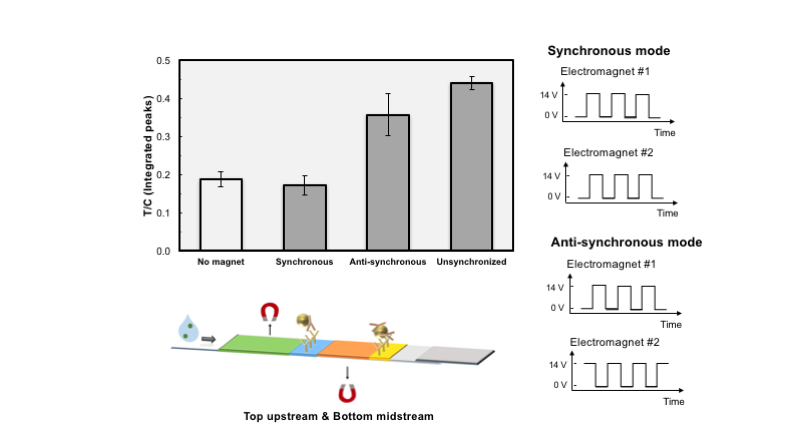


**S4 Fig. Effect of electromagnet operation mode on electromagnetically controlled LFA performance factor.** 13.5 ng/mL hCG was detected using anti-hCG antibodies at the test line and mouse monoclonal anti-β hCG antibodies-functionalized magnetic particles. Control line consisted of anti-mouse antibodies. Two electromagnets were applied in top upstream and bottom midstream positions with 10-second on/ 10-second off pulses at 14 V, in different operation modes (synchronous, anti-synchronous or unsynchronized). Line intensity profiles were evaluated by ImageJ density analysis. The area under each peak was numerically integrated using the ImageJ Gel Analysis Toolbox and the ratio of the intensity of the test line (T) divided by the intensity of the control line (C) for each strip was calculated and then averaged for replicate strips (n=3, mean ± SD).
