# Supplementary material for: Enhancement of lateral flow assay performance by electromagnetic relocation of reporter particles
Source: PLoS One. 2018 Jan 8;13(1):e0186782. doi: 10.1371/journal.pone.0186782 (PMC5757911; doi:10.1371/journal.pone.0186782)
Supplement: S5 Fig — (DOCX) [file pone.0186782.s005.docx]

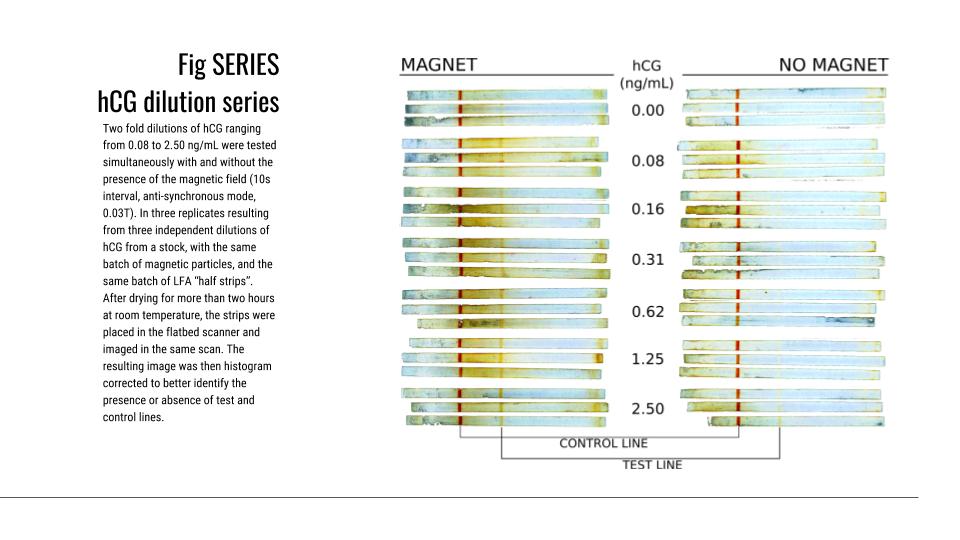


**S5 Fig. Effect of electromagnetically controlled LFA performance in hCG dilution series.** Two fold dilutions of hCG ranging from 0.08 to 2.50 ng/mL were tested simultaneously with and without the presence of the magnetic field (10s interval, anti-synchronous mode, 0.03T). In three replicates resulting from three independent dilutions of hCG from a stock, with the same batch of magnetic particles, and the same batch of LFA “half strips”. After drying for more than two hours at room temperature, the strips were placed in the flatbed scanner and imaged in the same scan. The resulting image was then histogram corrected to better identify the presence or absence of test and control lines.
