# Supplementary material for: Enhancement of lateral flow assay performance by electromagnetic relocation of reporter particles
Source: PLoS One. 2018 Jan 8;13(1):e0186782. doi: 10.1371/journal.pone.0186782 (PMC5757911; doi:10.1371/journal.pone.0186782)
Supplement: S6 Fig — (DOCX) [file pone.0186782.s006.docx]

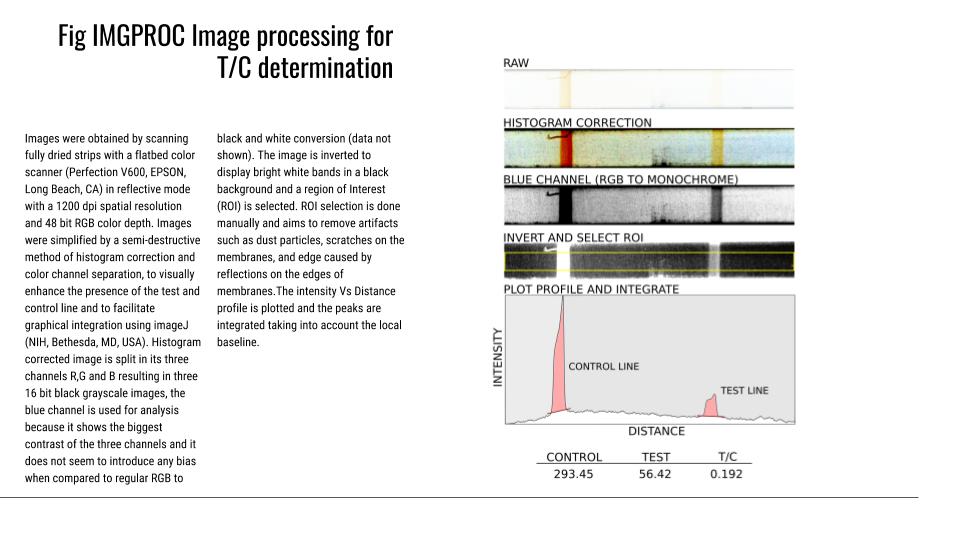

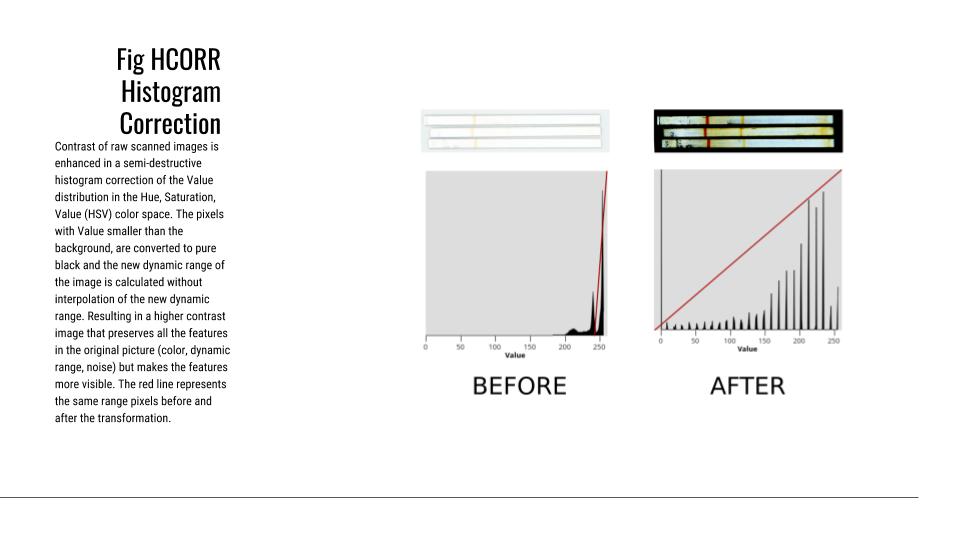


**S6 Fig. Image processing for T/C determination and histogram correction.** Images were obtained by scanning fully dried strips with a flatbed color scanner (Perfection V600, EPSON, Long Beach, CA) in reflective mode with a 1200 dpi spatial resolution and 48 bit RGB color depth. Images were simplified by a semi-destructive method of histogram correction and color channel separation, to visually enhance the presence of the test and control line and to facilitate graphical integration using ImageJ (NIH, Bethesda, MD, USA). Histogram corrected image is split in its three channels R,G and B resulting in three 16 bit black grayscale images, the blue channel is used for analysis because it shows the biggest contrast of the three channels and it does not seem to introduce any bias when compared to regular RGB to black and white conversion (data not shown). The image is inverted to display bright white bands in a black background and a region of Interest (ROI) is selected. ROI selection is done manually and aims to remove artifacts such as dust particles, scratches on the membranes, and edge caused by reflections on the edges of membranes. The intensity Vs Distance profile is plotted and the peaks are integrated taking into account the local baseline.
Contrast of raw scanned images is enhanced in a semi-destructive histogram correction of the Value distribution in the Hue, Saturation, Value (HSV) color space. The pixels with Value smaller than the background, are converted to pure black and the new dynamic range of the image is calculated without interpolation of the new dynamic range. Resulting in a higher contrast image that preserves all the features in the original picture (color, dynamic range, noise) but makes the features more visible. The red line represents the same range pixels before and after the transformation.
