# Supplementary material for: Enhancement of lateral flow assay performance by electromagnetic relocation of reporter particles
Source: PLoS One. 2018 Jan 8;13(1):e0186782. doi: 10.1371/journal.pone.0186782 (PMC5757911; doi:10.1371/journal.pone.0186782)
Supplement: S7 Fig — (DOCX) [file pone.0186782.s007.docx]

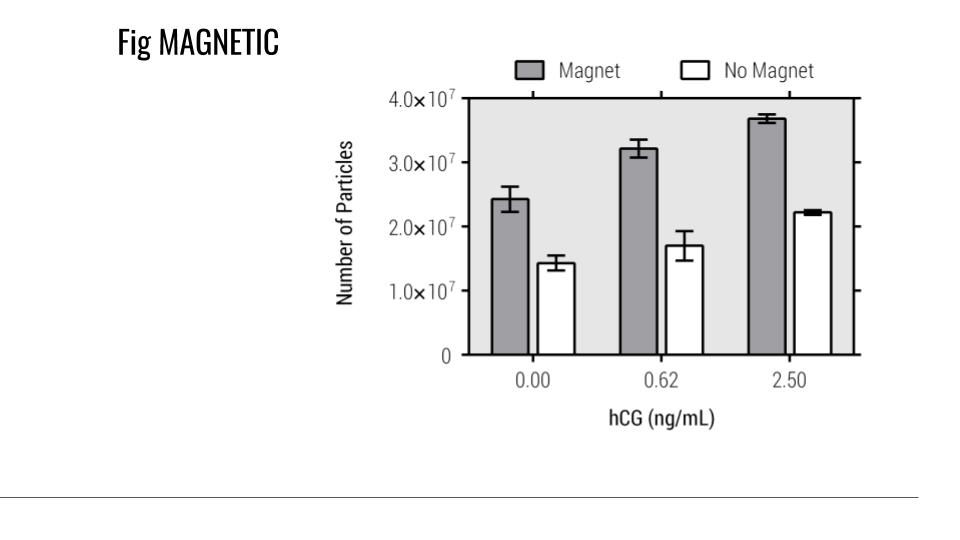

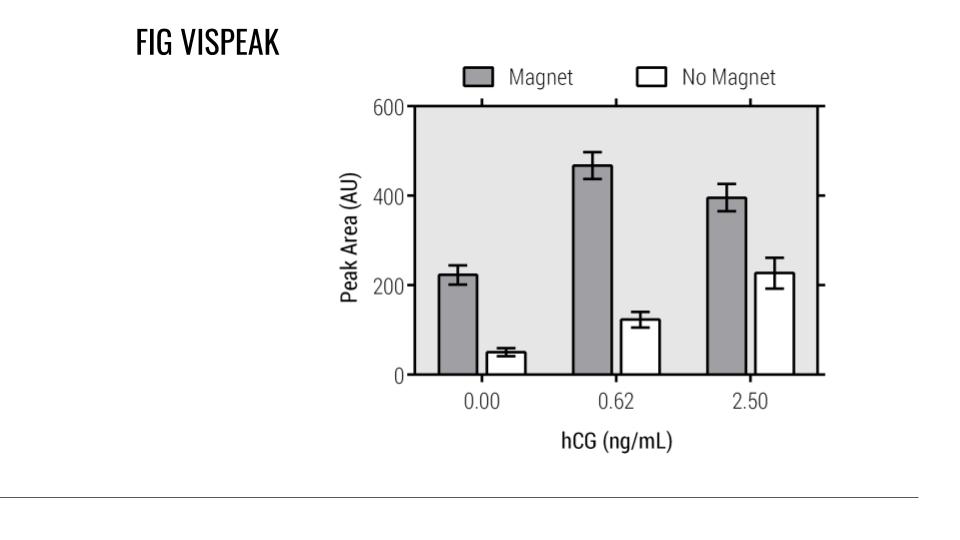


**S7 Fig. Number of particles and visual peak area at the test line for hCG dilution series in the presence and absence of the magnetic field.** Number of particles determined by AGFM. The magnetic properties of cut test lines were measured and the number of magnetic particles was determined for the concentrations of 0, 0.62 and 2.5 ng/mL hCG. (n=3). Mean and standard error are represented. Peak visual intensity determined in the 3x3mm cut out test line peak for the 0, 0.62 and 2.5 ng/mL hCG. Mean and Standard error are shown.
